# The Hepatitis E Virus Polyproline Region Is Involved in Viral Adaptation

**DOI:** 10.1371/journal.pone.0035974

**Published:** 2012-04-24

**Authors:** Michael A. Purdy, James Lara, Yury E. Khudyakov

**Affiliations:** Centers for Disease Control and Prevention, National Center for HIV/Hepatitis/STD/TB Prevention, Division of Viral Hepatitis, Atlanta, Georgia, United States of America; Institute of Infectious Disease and Molecular Medicine, South Africa

## Abstract

Genomes of hepatitis E virus (HEV), rubivirus and cutthroat virus (CTV) contain a region of high proline density and low amino acid (aa) complexity, named the polyproline region (PPR). In HEV genotypes 1, 3 and 4, it is the only region within the non-structural open reading frame (ORF1) with positive selection (4–10 codons with dN/dS>1). This region has the highest density of sites with homoplasy values >0.5. Genotypes 3 and 4 show ∼3-fold increase in homoplastic density (HD) in the PPR compared to any other region in ORF1, genotype 1 does not exhibit significant HD (p<0.0001). PPR sequence divergence was found to be 2-fold greater for HEV genotypes 3 and 4 than for genotype 1. The data suggest the PPR plays an important role in host-range adaptation. Although the PPR appears to be hypervariable and homoplastic, it retains as much phylogenetic signal as any other similar sized region in the ORF1, indicating that convergent evolution operates within the major HEV phylogenetic lineages. Analyses of sequence-based secondary structure and the tertiary structure identify PPR as an intrinsically disordered region (IDR), implicating its role in regulation of replication. The identified propensity for the disorder-to-order state transitions indicates the PPR is involved in protein-protein interactions. Furthermore, the PPR of all four HEV genotypes contains seven putative linear binding motifs for ligands involved in the regulation of a wide number of cellular signaling processes. Structure-based analysis of possible molecular functions of these motifs showed the PPR is prone to bind a wide variety of ligands. Collectively, these data suggest a role for the PPR in HEV adaptation. Particularly as an IDR, the PPR likely contributes to fine tuning of viral replication through protein-protein interactions and should be considered as a target for development of novel anti-viral drugs.

## Introduction

Hepatitis E virus (HEV), a hepevirus [1], causes epidemic and sporadic cases of hepatitis in humans [2]. Initially, HEV infection and hepatitis E were encountered primarily in developing countries, and in developed countries were recognized as associated with international travel. However, as detection techniques have improved, HEV infection has been found to be more prevalent than originally thought and is currently found worldwide [3]. HEV sequences are segregated into four genotypes. HEV genotypes 1 and 2 infect primarily humans along the fecal/oral transmission route, while genotypes 3 and 4 can infect humans, swine, deer and boar. In humans, infection by genotypes 3 and 4 appears to be primarily zoonotic [4], [5]. The epidemiology of HEV infections is complex and the virus can be transmitted through multiple modes [6].

The HEV genome is a positive-sense, single-stranded RNA of about 7.2 kb with a 5′-methylguanine cap and 3′-poly(A) tail. The genome contains three overlapping open reading frames (ORFs). ORF1 encodes the non-structural proteins responsible for viral replication. ORF2 encodes the viral capsid protein, and ORF3 encodes a protein, which has regulatory functions [1]. The ORF1 non-structural proteins share the highest homology with a group of viruses called rubi-like viruses which includes Rubivirus, Betatetravirus, Benyvirus, Omegatetravirus, *Sclerotinia sclerotiorum* debilitation-associated virus and cutthroat trout virus (CTV) [7], [8], [9].

Sequence divergence among HEV isolates is highest in a region preceding the helicase in the nonstructural polyprotein [10]. This region contains a disproportionate number of prolines as compared with the rest of the nonstructural polyprotein and is known as the polyproline region (PPR). Because of sequence variability the region is also known as the hypervariable region [11], [12]. The *Institut National de la Recherche Agronomique* (INRA), home of the Pfam database, states that the PPR belongs to protein family 12526 (DUF3729, CDD:152960) [13]. This protein is of unknown function and is found in association with several proteins including pfam 01660 (viral methyltransferase), pfam 05417 (protease C41), pfam 01661 (Appr-1″-p processing enzyme/macro domain) [14], pfam 01443 (UvrD/REP helicase) and pfam 00978 (RNA-dependent RNA polymerase). The HEV PPR was first reported by Koonin *et al.* [10] as a putative protein hinge. Proteins and peptides with stretches of multiple prolines may not have stable tertiary structure [15], [16], [17], which is consistent with the PPR acting as a hinge. However, the functions of the HEV PPR, if any, are unknown. The PPR does not appear to be required for the replication of rubivirus [18] or HEV [12]. Immediately upstream from the PPR is a region with no known function [10]. Tzeng *et al.* showed that a deletion of this region and part of the PPR created a rubivirus mutant that was unable to self-replicate [18]. However, Pudupakam *et al.* found that deletions in the PPR did not abolish HEV infectivity *in vivo* or *in vitro*, although near-complete deletion of the PPR yielded evidence of attenuation [12] and suggested that the PPR may interact with viral and host factors to modulate replication efficiency [19]. This paper presents data indicating a role of the PPR in HEV adaptation.

## Materials and Methods

The sequences used in this study were obtained from GenBank (Table S1).

### Sequence Alignment

HEV genotype 1 to 4 sequences were initially aligned with ClustalX (ver. 2) [20]. Peptides from the HEV PPR for genotypes 1, 3 and 4 were individually realigned using MUSCLE (ver. 3.6) [21].

### Identification of the PPR

To identify the PPR in sequences used in this study, a small Perl (Strawberry Perl 5.12.1.0) script was written to scan the viral genomes and determine the number of prolines within a 30-residue window and a step of one residue. A value of 9 prolines in a window was chosen as the cutoff for a PPR (cutoff was 8 for genotype 2).

### Shannon entropy

Shannon entropy was calculated for each position in an alignment of amino acid (aa) sequences using BioEdit (ver. 7.0.5.3) [22]. Because of the per residue variation, the average entropy for a sliding window of 30 residues with a one-residue step was used to smooth the data for this analysis.

### Selective pressure

Selective pressure was calculated as *dN/dS* using the one-rate fixed effects likelihood method in HyPhy (ver. 2.0020101222beta) with a p<0.05 considered statistically significant [23].

### Homoplastic density

The homoplasy index was calculated using PAUP*(ver. 4.10beta) with the apolist command [24]. The homoplastic density was obtained by counting the number of homoplastic sites with values >0.5 within a 50-nucleotide (nt) sliding window with a one-base step.

### Intrinsically disordered region prediction

Intrinsically disordered regions (IDRs) in individual sequences were predicted using the DISOPRED2 server [25] at its default settings. This method was chosen because it is conservative in its estimations of disorder. DISOPRED2 is based on a database of high-resolution crystallographic information (≤2 Å). A sequence query is aligned to similar regions from Protein Data Bank (PDB: www.pdb.org) for which no coordinate information is available, as disordered regions cannot be modeled through crystallography. These results were confirmed using IUPred by screening for long disordered regions [26]. IUPRED is based on the computation of pair-wise interaction energies of residues in the query sequences to estimate their tendency to form stabilizing pair-wise interactions [27].

### Linear Motifs

Functional sites in the PPR conforming to the constraints of linear motifs (LMs) [28], [29] were predicted using the Eukaryote linear motif (ELM) server at elm.eu.org [30]. LMs were predicted for each genotype (genotypes 1–4) and the Japanese wild boar sequences [31], and compared to identify motifs found in all sequences. Only LMs that were found to be common to all the sequences were considered to be putative HEV LMs. For example, the LIG_CYCLIN_1 site was discarded from this analysis because a required MOD_CDK motif was found only in genotype 3 sequences.

### Tree comparison

Continuous non-overlapping windows in HEV genotypes 1, 3 and 4 were created using the length of the polyproline region for each genotype as the window size. The windows for each genotype were situated so that one of the windows would be the polyproline region itself. Neighbor-joining trees were created for aligned full-length ORF1 sequences and each of the window regions with DNADIST, using the Kimura 2-parameter substitution model, and NEIGHBOR [32]. The tree for each window region was compared to the full-length ORF1 tree with the nodal and split distance methods in TPOD/FMTS (ver. 3.3) [33].

### 3-Dimensional (3D) Structure

Analysis was conducted using the PPR of the HEV genotype 3 sequence with GenBank accession number AB091394. The PPR was 81 residues long located between protein positions 707–831 (according to reference M74506).

Ten full-atomic *ab-initio*-based 3D models of the HEV genotype 3 PPR were automatically generated using the I-TASSER software package (ver. 1.1) [34], [35]. Briefly, to excise continuous fragments from template alignments, 5 threading programs were sequentially implemented against the PDB to select the best templates matching the query sequence. Template selection by each method was restricted to 20 matches. Assembly of the continuous fragments was performed by running 14 Monte Carlo simulations. Full-atomic 3D models were generated after energy minimization refinements of assembled structures. Accuracy of predicted models was evaluated by confidence scores (C-scores). The C-score is a confidence score for estimating the quality of predicted models. This score is typically in the range of [−5, 2], where a high value signifies a model with a high confidence and vice-versa. A TM-score >0.5 indicates a model of correct topology and a TM-score <0.17 means a random similarity. A full detail of implemented protocols is available in [34].

Molecular analysis described herein was performed using the top-ranked 3D model. To assess the overall stereochemical quality of the generated 3D model, the geometrical accuracy of the residues and 3D profile quality index were inspected with the PROCHECK (ver. 3.5) [36] and VADAR (ver. 1.8) [37] programs, respectively. Additional refinement to remove atomic clashes was carried out using the WHATIF (ver. 8.0) modeling package software [38].

### Secondary Structure

The sequence-based prediction of the secondary structure of the HEV PPR, was performed using PSIPRED (ver. 2.6) [39]. Secondary structure assignment in the PPR was done using the standard DSSP method [40].

### Accessible Molecular Surface

To assess the exposed surface area, the accessible surface area (ASA) or the area where a water molecule could access or contact, the accessible molecular surface of residues in the PPR model was calculated. The ASA for each residue was computed and measured in square Angstroms (Å) as implemented in the WHATIF modeling package software [38].

### Electrostatic Surface

Simulation of atomic-scale information on energetic contributions to atomic interactions and biomolecular structure of the HEV PPR 3D model was performed to examine possible biomolecular functions and highlight regions of potential interest. Electrostatic properties were evaluated via an implicit method for modeling biomolecular solvation through solution of the Poisson-Boltzmann (PB) equation. The electrostatic potentials around the PPR molecule were calculated with APBS (ver. 1.2) [41] and visualized with PyMOL (ver. 1.1r2pre) [42].

### Structure-based function predictions

To associate possible molecular functions to the PPR 3D model, gene ontology (GO) terms were derived using a structure-based method for annotation of biological function. The 3D model was threaded against a set of function libraries by global and local structure matches. GO terms were then derived from the best functional homology templates (TM score cutoff >0.5). A total of ten matches were extracted and ranked by their TM-score [34], [35]. GO terms associated with each of these functional PDB template analogs were compiled and the consensus GO terms among them was used to predict molecular functions in the PPR. Analysis was conducted using the COFACTOR algorithm [43], [44].

### Protein binding regions in IDRs

Disordered protein segments can function as linkers between globular domains because of their flexibility [45]. Additionally, disordered regions in proteins can have important functional roles by binding specifically to other proteins, DNA or RNA ligands. These binding interactions, known as coupled folding and binding, involve transient disorder-to-order state transitions with more stable secondary and tertiary structural elements [46], [47]. Studies have shown that disordered regions with the properties to be involved in coupled folding and binding can be distinguished from those that lack them [48], [49], [50], [51].

The PPR IDR was examined to determine its ability to undergo coupled folding and binding using an algorithm to identify protein binding regions. Predictions were carried out using the ANCHOR program (ver. 1.0) [52], [53]. Briefly, prediction of protein binding regions was done from the aa sequence of PPR (ref. AB091394). Recognition of binding regions was based on the properties of residues located in the IDR to energetically gain favorable intra-chain interactions to form a stable secondary structure by interacting with a protein partner. Estimation of residue properties relied on the energy estimation framework implemented for disorder predictions in IUPRED [27]. To decrease the false-positive rate on globular domains and transmembrane segments, short regions <6 residues and regions with an average IUPRED score (disorder tendency of a residue) <0.1 were filtered out.

## Results

### Proline distribution within the ORF1-protein

The ORF1-encoded proteins of hepeviruses were examined using a Perl script to count the number of Pro residues within a 30-aa sliding window. Analysis identified protein regions with the highest Pro density across this family of viruses (Table 1). An alignment of HEV genotype 1–4 sequences showed that the PPR was bound by conserved sequences TLYTRTWS and RRLLXTYPDG at the N- and C-sides, respectively. HEV isolated from avians and rats were also found to have PPR, but did not have the conserved sequence boundaries found in genotypes 1–4. Sequence conservation of PPR boundaries could not be established with certainty because of the limited number of extant sequences. Similar to genotypes 1–4, the PPR in the avian and rat sequences was located upstream from the putative macro domain as shown earlier [14].

**Table 1:** Calculated boundaries for viral IDRs and highest proline density.

		IDR			Polyproline			
ID		start	stop	max	start	stop	Pro	avg P
AY535004	Av	533	618	0.845	560	562	19	18.8
GU345042	Ra	570	678	0.612	595	624	22	22.5
M80581	1	711	778	0.604	729	754	18	16.6
M74506	2	701	717	0.211	759	760	14	
AB369691	3b	720	788	0.748	729	781	27	27.0
EU723515	3f	710	818	0.641	758	810	35	27.0
AB220972	4	710	795	0.682	726	747	22	20.5
AB602441	WB	711	799	0.641	770	771	20	17.5
DQ085338	Rub	713	808	0.943	730	770	26	26.0
HQ731075	CTV	637	725	0.577	710	712	19	

ID shows GenBank accession numbers for sequences used in the IDR calculation (DISOPRED2 [25]) and the polyproline sliding-window analysis. Class codes to right of each ID are: Av, avian; Ra, rat; genotype 1; genotype 2; subgenotype 3b; subgenotype 3f; genotype 4; WB, Japanese wild boar; Rub, rubivirus and CTV, cutthroat trout virus. The start and stop positions are those obtained from each calculation. Max is the maximum disorder probability in the PPR (threshold = 0.05). Pro lists the number of prolines in each PPR IDR. Avg P is the average number of prolines in all members of a class. Empty avg P indicates presence of only a single member in that class.

### Sequence diversity

**Figure 1:**
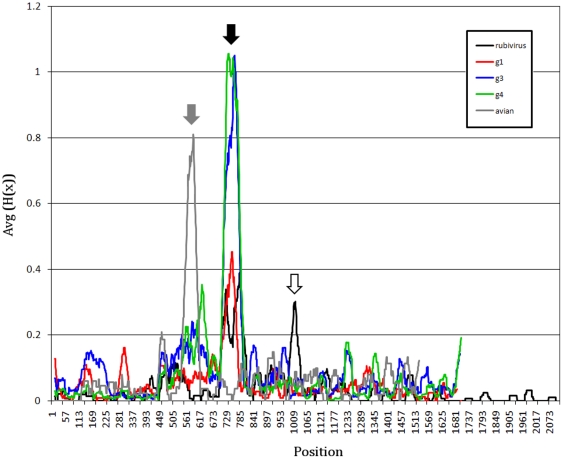
Shannon entropy for alignments of rubi-like viruses. Data are shown as the average Shannon entropy in 30-aa acid windows with a one-residue step. Sequences are full-length with the three ORFs concatenated in head-to-tail fashion as ORF1, ORF2 and ORF3. Rubi (rubivirus), DQ085338; g1 (genotype 1), M80581; g2 (genotype 2), M74506; g3 (genotype 3), AB369691; g4 (genotype 4), AB220972; avian, AY535004. Subtype 3f sequences have a 27-aa sequence duplication removed from the PPR to allow better alignment of sequences. The PPRs are located by the grey arrow (avian HEV) and the black arrow (rubivirus and HEV genotypes 1, 3 and 4). The white arrow is immediately upstream from the rubivirus endopeptidase (centered near residue 1010).

Shannon entropy analysis using a 30-aa sliding window showed that the PPR in human and avian HEV represents the most divergent protein region (Fig. 1). The PPR sequence divergence is >2-fold greater for HEV zoonotic genotypes 3 and 4 than for human genotype 1, suggesting the potential association between sequence heterogeneity and the number of hosts.

### Selection

**Figure 2:**
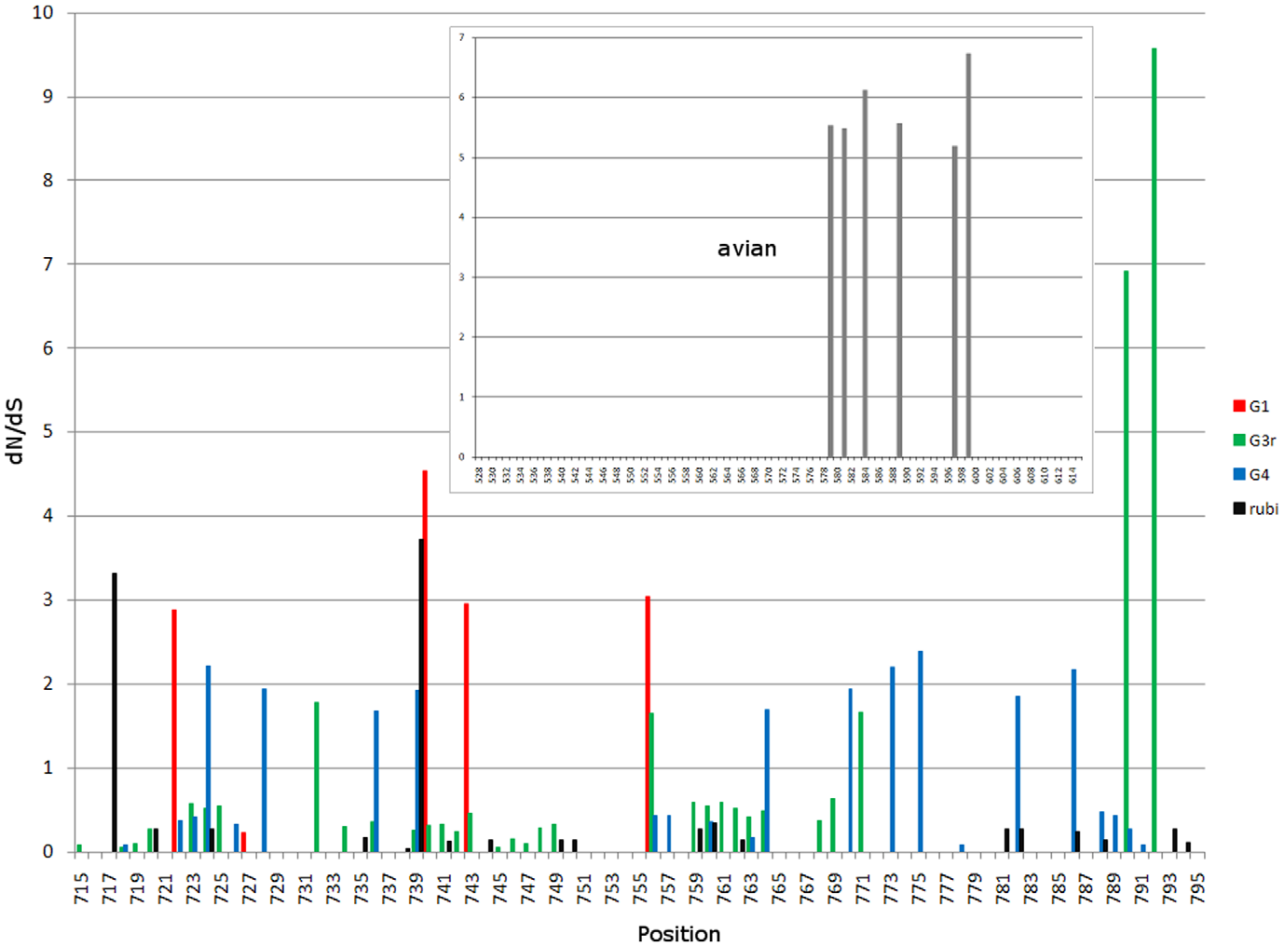
*dN/dS* values for rubivirus and HEV. For genotypes 1, 3 and 4, and rubivirus, results of analysis are shown across from aa positions 701 to 800, which includes the PPR IDR region. Values >1 represent positions under positive selection. Insert shows *dN/dS* values for the PPR IDR of avian HEV (528 to 614 aa). There are no *dN/dS* values ≥1 in ORF1 outside the regions shown.

An examination of selective pressure along ORF1 was conducted using calculation of *dN/dS* values for each codon. It was found that ORF1 is in general under strong negative selection. There are no *dN/dS* values with ≥1 anywhere in ORF1 except for the region encoding for the PPR. The PPR from genotypes 1, 3 and 4 contains 4–10 codons with *dN/dS*>1, indicating that the PPR is the only genomic region in these genotypes that has experienced detectable positive selection (Fig. 2).

### Homoplastic density

**Figure 3:**
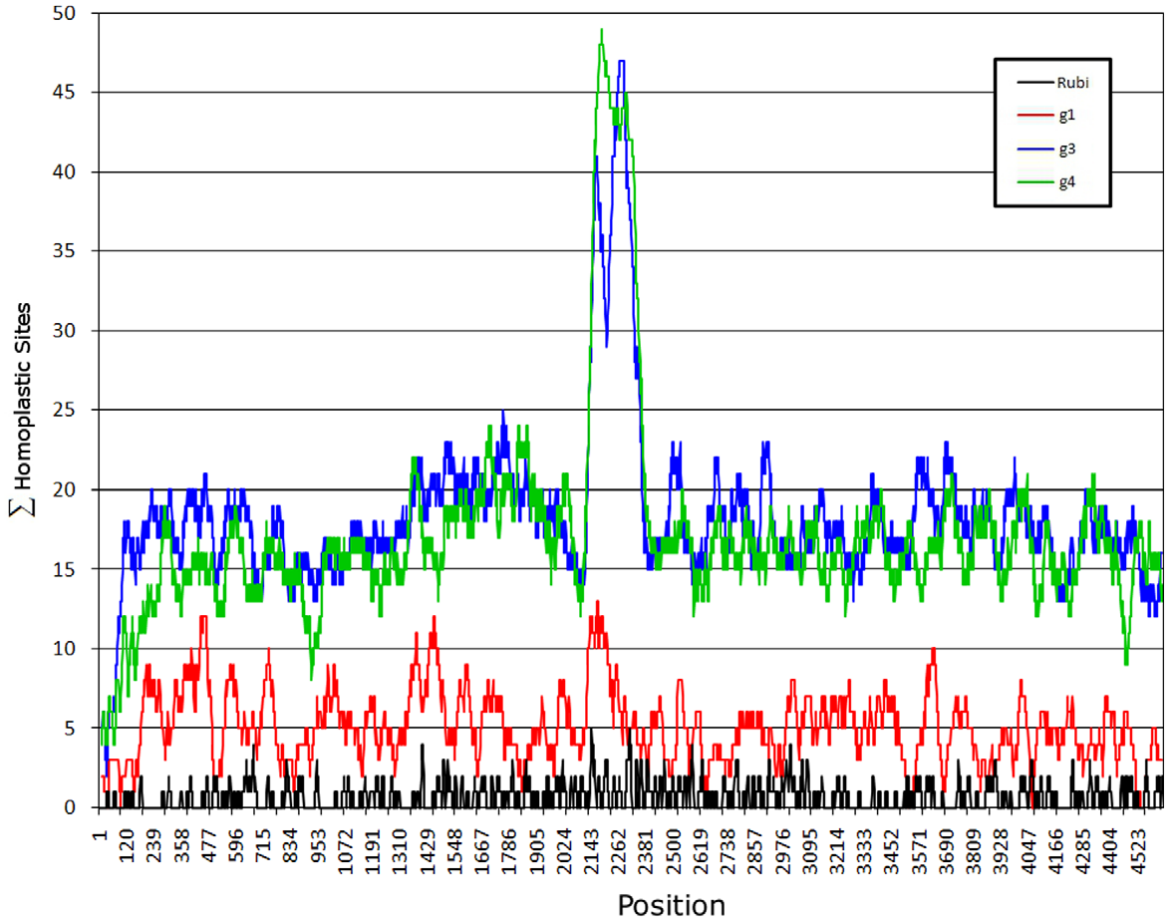
Homoplastic density. A sliding window was used to count the number of aa within each window having a homoplastic index of ≥0.5 (shown on the left). Numerals below y axis represent nt positions in ORF1.

The PPR has the highest density of sites with homoplasy index values >0.5 (Fig. 3). Genotypes 3 and 4 show ∼3-fold increase in density of strongly homoplastic sites (HD) in the PPR compared to any other region in ORF1 whereas genotype 1 does not exhibit significant HD (p<0.0001) (Fig. 3). In general, the genotype 3 and 4 ORF1 has a ∼2-fold greater HD than genotype 1. The genotype 1 sequence database contains only 17 sequences, whereas the genotype 3 and 4 databases contain 74 and 55 sequences, respectively. To eliminate the possibility that the observation of a lower HD in genotype 1 is related to a small number of sequences available, 17 sequences were selected at random for genotypes 3 and 4. However, analysis of the lower number of sequences still yielded high HD in the PPR (data not shown). Additional analysis was conducted using only the genotype 3b sequences alone (n = 20). The genotype 3b PPR shows a high HD, but is 30% lower than seen with all genotype 3 or 4 sequences (data not shown). The data suggest that the higher HD in genotypes 3 and 4 result from a greater sequence divergence as compared to genotype 1, possibly reflecting the more diverse host range for genotypes 3 and 4.

### The intrinsically disordered region

Many polyproline regions are known to be unstructured or disordered [54]. To examine whether the HEV PPR belongs to this class of IDRs, 2 algorithms were used in this analysis. The HEV genotype 1, 3 and 4 PPRs were predicted to be IDRs located between conserved sequences TLYTRTWS and RRLLXTYPDG. Genotype 2 was also predicted to have an IDR, located between these conserved sequences, which, however, does not coincide with the region of the highest Pro density as was observed for genotypes 1, 3 and 4 (Table 1). This table also shows that the putative genotype 2 PPR has the lowest probability of being an IDR and the lowest number of prolines.

**Figure 4:**
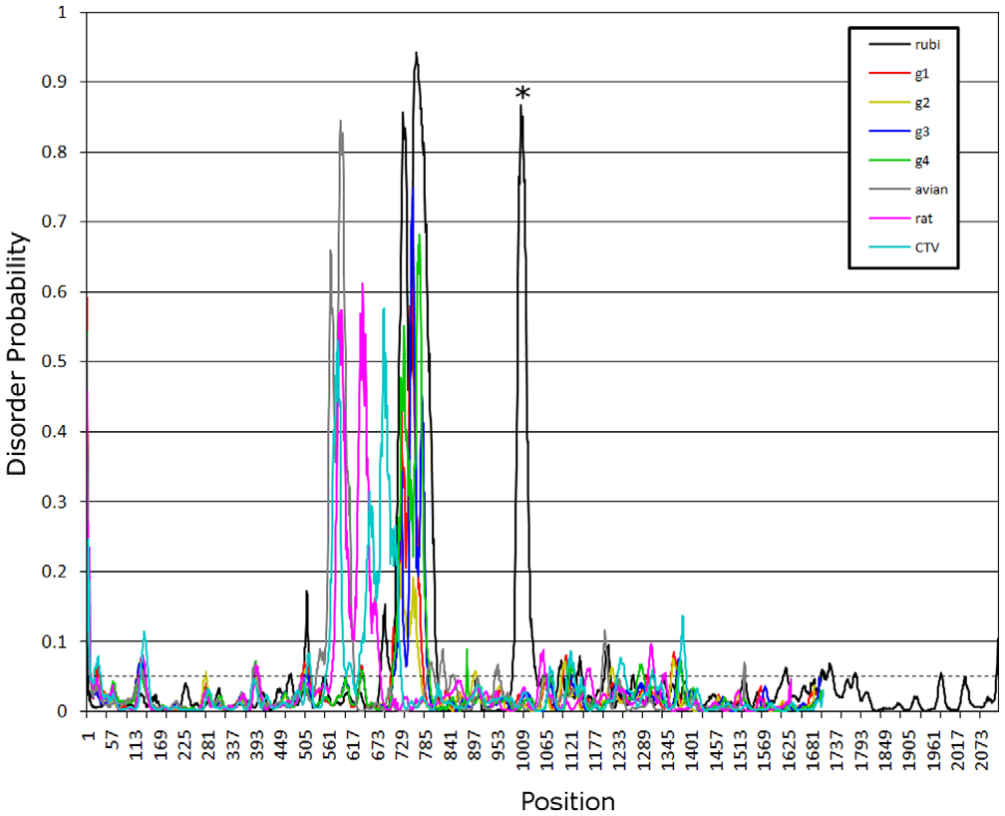
Disorder probability. Disorder probability for aa sequence of selected sequences was calculated using DISOPRED2. A threshold value of 0.05 was set to distinguish between ordered and disordered region along the genome (dashed line). Regions above the threshold are predicted to be disordered (see table 1). All these viruses have a peak above 0.05 within their respective PPRs, positions 570 to 800. Rubivirus has two peaks, one in the PPR and the second at about position 1000, which is just upstream of the endopeptidase (denoted by the asterisk).

The PPRs in avian and rat HEV located upstream from the putative macro domain were also predicted to be IDRs (Fig. 4). Taking into consideration that the predicted genotype 1–4 IDRs are flanked by or located near conserved regions, the conserved IDR boundaries may be located between aa positions 533 and 618 (AY535004), and 570 and 678 (GU345042) of the ORF1-encoded polyprotein for avian and rat HEV, respectively. An alignment of CDD:152960 sequences at NCBI [55] suggests that the conserved aa sequences KLLTLKELA and EEVLALLP [13] in avian HEV serve as the N- and C-terminal IDR boundaries.

**Figure 5:**
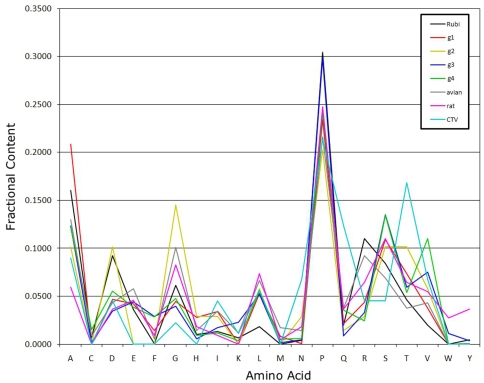
Proportion of aa in the PPR IDR of rubi-like viruses.

The PPRs from genotypes 1, 3 and 4, and avian HEV contain a low fraction of Ile, Met, Phe, Trp and Tyr and a high fraction of Ala, Gly, Pro and Ser (Fig. 5), which is consistent with a low aa complexity of known IDRs. IDRs usually have a low proportion of bulky hydrophobic aa, a high proportion of polar and charged aa [45] and structure breaking aa, like Pro and Gly [27].

**Figure 6:**
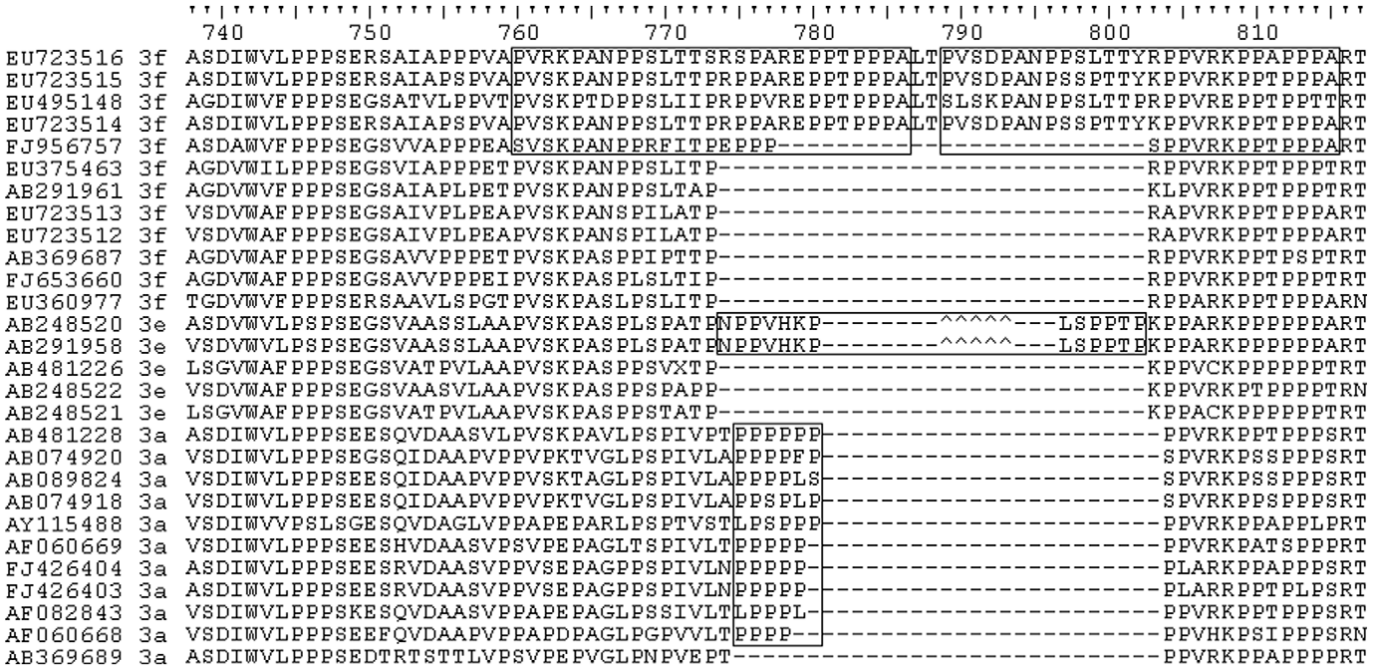
Sequence duplications in genotype 3. Selected genotype 3 PPR sequences were aligned against EU723516. Each sequence is identified by its GenBank accession number and subtype. Boxes show sequence duplications for 3f and insertions for 3e and 3a sequences. Carets identify an alternative alignment region for the PVHKP peptide (positions 776–780) for genotype 3e.

Duplications of sequence have been found within some IDRs [15], [54]. An examination of genotype 3 PPR sequences revealed that some genotype 3f sequences contain a 27-aa, head-to-tail duplication (Fig. 6) near the C- terminus of the PPR. Further examination showed that some genotype 3e sequences contain a potentially more complex indel pattern, which results in a 13-aa insertion in this region and some genotype 3a sequences have a 4- to 6-residue insertion in this region dominated by Pro (Fig. 6).

### PPR of rubi-like viruses

Homology between HEV and rubivirus non-structural genes suggests that HEV belongs to a group of animal, plant and mycotic viruses known as the rubi-like viruses [7]. Besides hepeviruses, only rubiviruses and CTV contain a PPR located upstream from the macro domain (Table 1). The rubivirus PPR also has high genetic diversity (Fig. 1) and sites under positive selection (Fig. 2). However, similar to genotype 1, the rubivirus PPR as well as the entire non-structural polyprotein has a very low HD (Fig. 3). CTV could not be tested for these properties as there is only one full-length sequence extant [9]. Like the hepeviruses, rubiviruses and CTV exhibits high disorder probability in its PPR (Fig. 4). The rubivirus PPR also has a low aa complexity (Fig. 5).

### Phylogenetic fidelity

**Figure 7:**
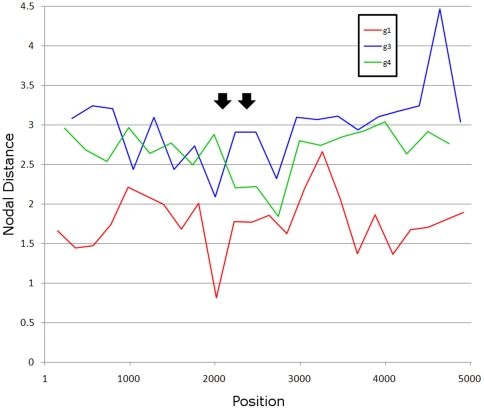
Nodal distance. The nodal distance calculated for consecutive non-overlapping windows in ORF1. The closer to zero the nodal distance is for a window, the more it is like the nodal distance for the full-length ORF1. Black arrows delineate the PPR.

The detection of considerable homoplasy and high genetic diversity in the PPR, as compared to other regions in the ORF1, suggests that there should be a substantial decline in homology among HEV sequences in the PPR, particularly for genotypes 3 and 4 sequences. This decline should be reflected in distortion of phylogenetic relationships among different sequences in the PPR. However, such a suggestion contrasts to the successful use of phylogenetic trees to genotype HEV isolates using this region [56], [57], [58], [59]. To determine whether the PPR phylogenetic signal is reduced when compared to other ORF1-regions, the TOPD/FMTS program [33] was used to examine concordance of phylogenetic trees generated using a sliding window across the ORF1. The phylogenetic signal for the tree from each window was compared to a tree for the entire ORF1. Using the nodal distance method, the PPR was found to have about the same phylogenetic signal as any other region when compared to the complete ORF1 tree (Fig. 7).

### Linear Motifs

Linear motifs (LMs) are short peptide segments that do not require 3D organization in order to function. These motifs operate as sites of regulation and are found in IDRs [28], [29]. Accordingly, the HEV PPR was examined for the occurrence of LMs. Using the search engine at elm.eu.org, seven LMs were found to be common across all four HEV genotypes and in HEV sequences from Japanese wild boars (Table 2). These sites, which are all in the IDR, included two protease cleavage sites (CLV_NDR_NDR_1 and CLV_PCSK_SKI1_1), three ligand binding sites (LIG_EH_1, LIG_EVH1_1 and LIG_SH2_STAT5) and two kinase phosphorylation sites (MOD_PKA_2 and MOD_PLK).

**Table 2:** List of ELM's common to HEV genotypes 1–4 in the PPR.

Motif	Mod	Description	Notes	pfam ID
CLV_NDR_NDR_1		N-Arg dibasic convertase (nardilysine) cleavage site	N-arginine dibasic convertase is an endopeptidase in dibasic sites processing secreted proteins.	PF00675
CLV_PCSK_SKI1_1		Subtilisin/kexin isozyme-1 (SKI1) cleavage site	The subtilisin-like proprotein convertases are expressed in mammalian neural and endocrine cells and play a major role in the proteolytic processing of both neuropeptide and peptide hormone precursors.	PF00082
LIG_EH_1		Asn-Pro-Phe motif responsible for the interaction with Eps15 homology (EH) domain	NPF motif interacting with EH domains, usually during regulation of endocytotic processes and vesicular trafficking	
LIG_EVH1_1		Proline-rich sequences that bind to the signal transduction modules EVH1	Many EVH1-containing proteins are associated closely with actin-based structures and are involved in re-organization of the actin cytoskeleton. The engrailed homology domain 1 motif is found in homeodomain containing active repressors and other transcription families.	
LIG_SH2_STAT5	**X**	STAT5 Src Homology 2 (SH2) domain binding motif.	STAT5 Src Homology 2 (SH2) domain binding motif. This is one of the most promiscuous motifs in ELM. It will match to approximately every third Tyr residue. Therefore the predictive power is very weak.	PF00017
MOD_PKA_2	**X**	Secondary preference for PKA-type AGC kinase phosphorylation.	PKA belongs to the large set of related AGC kinases having a preference for phosphorylating basophilic sites. cAMP-dependent protein kinase A (PKA) is the major target for cAMP action in eukaryotic cells.	
MOD_PLK	**X**	Site phosphorylated by the Polo-like-kinase	Site recognised and phosphorylated by the Polo-like-Kinase	PF00659

Motif is the ELM motif. X in the Mod column indicates phosphorylation of Thr/Ser in the motif. Pfam ID contains the ID for the protein family that cleaves or modifies the motif.

### Protein binding region in the HEV genotype 3 PPR

**Figure 8:**
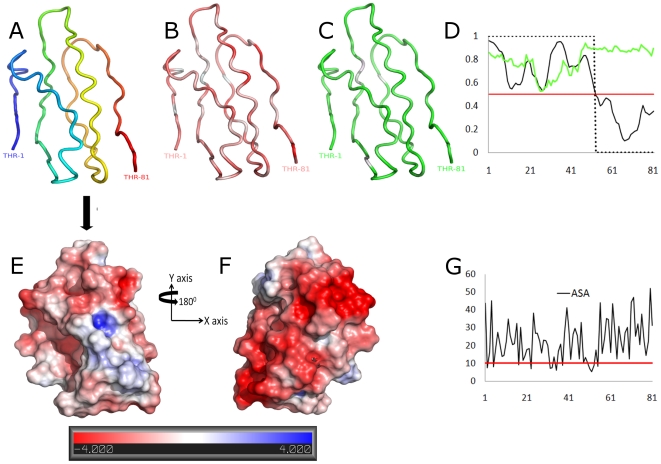
Predicted tertiary structure HEV genotype 3 PPR. (A) Secondary structure features of the 3D model; coloring is based on transition from N-termini (in blue) to C-termini (red). (B) Distribution of hydrophobicity, hydrophilic regions shown with red and hydrophobic with white (based on a normalized consensus aa scale [64]). (C) Degree of flexibility; flexible regions shown in green and rigid with white (based on an aa scale [65]). (D) Prediction of transient disorder-order binding region (based on the ANCHOR program); y axis represents probability scores and the x axis the residue positions. Green line - disorder tendency, solid black line - disorder-order tendency, dot line - binding score and red line - threshold. (E ) Electrostatic potential is mapped onto the modeled surface, colored by potential for solvent accessible surface (at a threshold level between −4 and 4). Negative and positive potentials indicated in red and blue, respectively. (G) Surface accessibility plot; y axis represents the total accessibility in squared Å and the x axis the aa position. A minimum area of 10 Å^2^ is needed to dock a water molecule (red line; accessibility threshold). 3D rendering was done in PyMOL [42].

The ANCHOR algorithm was used to show that the 52-aa region in the PPR (AB091394) between aa positions 707–758 (ref. M74506) is prone to transient disorder-to-order transitions upon binding to a protein ligand. The highest probability scores (≥0.74) were observed for subregions 707-TSGFSSDFS-715 and 737-VSDIWVLPP-745 (Fig. 8D). Four of the LMs were found to be located within these subregions, namely, kinase phosphorylation sites (MOD_CK1_1, 708-SGFSSDF-714 and MOD_GSK3_1, 708-SGFSSDFS-715), glycosaminoglycan attachment site (MOD_GlcNHglycan, 707-TSGF-710), and ligand binding site (LIG_SH3_3, 739-DIWVLPP-745).

### 3D-model of the HEV genotype 3 PPR

To examine further the structural properties of the PPR and investigate its possible molecular functional roles, we predicted the 3D molecular structure of the HEV PPR. The top-ranked 3D-model generated by I-TASSER [34], [35] yielded a C-score = −2.71, an estimated accuracy of 0.4±0.14 (TM-score) and an estimated resolution of 9.4±4.6 Å. Identification of 98.1% of residues falling within favorable and allowed regions of the Ramachandran plots and analysis of the 3D-profile indexes (Fig. S1 and S2) indicate a good stereochemical quality of the 3D-model (Fig. 8E). Lack of regular strand and helix secondary structure was observed in the PPR model generated by the DSSP secondary structure assignment [40]. Based on PSIPRED's 3-state, secondary structure prediction for this region, all residues were identified as random coil (Fig. S3). Also shown in Fig. 8 are aa physicochemical properties mapped onto the 3D model, which reveal the PPR's high composition of flexible and hydrophilic polar residues. These findings provide evidence that this PPR is intrinsically disordered.

### Surface and electrostatic properties

Approximately 70% of the residues in the PPR 3D-model were observed to be exposed (surface accessibility ≥15 Å^2^; Fig. 8G). Mapping of the computed electrostatic potentials onto the surface showed that the PPR surface is mainly negatively charged (Fig. 8E). Two major regions (surface patches) of high negative polarity (indicated by color density in Fig. 8E) were identified on the surface of PPR. The electrostatic potential properties at these two sites suggest that the PPR may be involved in protein-protein interactions [60].

### Prediction of molecular functions

COFACTOR was used to identify putative molecular functions of the PPR based on the predicted 3D structure by using GO annotations [43], [44]. The best PDB structural analogs of the PPR and the consensus GO annotations associated to them are shown in Table 3. Binding interactions were found to be the major molecular functional role among the top six functions attributed to the PPR, of which protein-protein interaction was one of such roles (GO:0005515; in Table 4). These results suggest that, similar to other IDRs [45], the PPR may be involved in binding of a wide variety of substrates.

**Table 3:** Predicted molecular functions for the HEV PPR model.

Rank	TM-Score	RMSD	Identity	Coverage	PDB homolog	Molecular function GO terms
1	0.5528	3.35	0.04	0.95	3eqnA	0004338, 0016787, 0016798, 0008152
2	0.5385	3.62	0.15	0.95	3l3sB	0016853, 0003824, 0008152
3	0.5374	3.37	0.04	0.93	2iq7A	0008152, 0007047, 0004650, 0016787, 0016798, 0005975
4	0.5373	3.64	0.03	0.91	1wmrA	0051675, 0008152, 0016798, 0005576, 0016787, 0004553
5	0.533	3.5	0.01	0.93	1k5cA	0004650, 0005975
6	0.5316	3.41	0.13	0.91	1k5dC	0000070, 0005098, 0006913, 0005096, 0048471, 0051383, 0043547, 0031965, 0005829, 0030702, 0031291, 0032853, 0005737
7	0.5305	3.47	0.05	0.91	1nhcA	0004650, 0005576, 0016787, 0016798, 0007047, 0008152, 0005975
8	0.5289	3.59	0.05	0.93	3p85A	0003824, 0008152
9	0.5285	3.62	0.11	0.93	1vrgA	0046872, 0016874
10	0.5276	3.4	0.08	0.9	3gf7A	0016874

TM-score is a measure of global structural similarity between query and template protein. RMSD is root mean standard deviation between residues that are structurally aligned by TM-align [66]. Identity is percentage sequence identity in the structurally aligned region. Coverage represents coverage of global structural alignment and is equal to the number of structurally aligned residues divided by length of the query protein. PDB analog is the PDB-matched template or functional analog, from which associated GO terms was used to predict function of query sequence.

**Table 4:** Molecular function of predicted consensus GO terms for the PPR model.

Consensus GO terms	Molecular function description
GO:0003824	Enzyme activity. Enzymes possess specific binding sites for substrates, and are usually composed wholly or largely of protein.
GO:0005488	Ligand. The selective, non-covalent, often stoichiometric, interaction of a molecule with one or more specific sites on another molecule.
GO:0000166	Interacting selectively and non-covalently with a nucleotide, any compound consisting of a nucleoside that is esterified with (ortho)phosphate or an oligophosphate at any hydroxyl group on the ribose or deoxyribose.
GO:0005524	Interacting selectively and non-covalently with ATP, adenosine 5′-triphosphate, a universally important coenzyme and enzyme regulator.
GO:0016874	Catalysis of the joining of two substances, or two groups within a single molecule, with the concomitant hydrolysis of the diphosphate bond in ATP or a similar triphosphate.
GO:0005515	Interacting selectively and non-covalently with any protein or protein complex (a complex of two or more proteins that may include other nonprotein molecules).
GO:0016787	Catalysis of the hydrolysis of various bonds, e.g. C-O, C-N, C-C, phosphoric anhydride bonds, etc. Hydrolase is the systematic name for any enzyme of EC class 3.
GO:0003989	Catalysis of the reaction: ATP+acetyl-CoA+HCO3− = ADP+phosphate+malonyl-CoA.
GO:0004650	Catalysis of the random hydrolysis of (1→4)-alpha-D-galactosiduronic linkages in pectate and other galacturonans.
GO:0046872	Interacting selectively and non-covalently with any metal ion.

Consensus GO terms in ranked order. Ranking based on GO scores [43], [44].

## Discussion

The functional significance of the HEV PPR remains unknown. The data obtained in the present study suggest that the substantial sequence variability of PPR plays an important role in viral adaptation. Although the exact role of the PPR in HEV adaptation is not known, several findings indicate that the PPR may be involved in determination of host range. The HEV lineages of genotypes 1 and 2 are anthropotropic, while the genotype 3 and 4 HEV strains infect not only humans but also several animal species [5], suggesting their zoonotic origin. Consistent with the broad host range of zoonotic HEV lineages, PPR in HEV genotypes 3 and 4 is ∼2-fold more heterogeneous than in HEV genotype 1 (Fig. 1). The limited number of available sequences did not allow for assessing the degree of PPR heterogeneity in genotype 2.

Analysis of distribution of highly homoplastic sites along ORF1 was especially informative (Fig. 3). It was found that the PPR had the 3-fold greater HD than any other region within ORF1 of HEV genotypes 3 and 4, although no difference in HD was observed along ORF1 in genotype 1. Moreover, Shannon entropy in ORF1 from genotypes 3 and 4 was >2 times greater than in genotype 1 in the PPR (Fig. 1). The presence of many highly homoplastic sites indicates the operation of recurrent selection pressures on ORF1 and especially the PPR in the zoonotic genotypes 3 and 4. This finding suggests convergent evolution of the PPR, which is probably related to shuttling HEV infection among various susceptible species of hosts.

The PPR is the only region in ORF1 that contains sites under positive selection (Fig. 2). All 3 HEV genotypes, for which the sufficient number of sequences was available for analysis, contain 4–10 codons with *dN/dS*>1. Since both anthropotropic and zoonotic genotypes experienced detectable positive selection, the PPR is not only involved in adaptation to the host range but expresses other adaptive traits shared by all genotypes.

The PPR also has a low content of bulky hydrophobic aas (Ile, Met, Phe, Trp and Tyr) and a high proportion of polar and charged aas (Ala, Gly, Pro and Ser) (Fig. 5) [27], [45]. The fractional content of aa in the PPR is maintained across all four HEV genotypes (Fig. 7). The confluence of findings showing high Pro density, high average Shannon entropy (Fig. 1), positive selection (Fig. 2) and reduction in ordered secondary structure (Fig. 4) strongly suggests that the HEV PPR is an IDR. Analysis carried out on the PPR 3D-model for HEV genotype 3 also confirmed that the PPR lacks regular structure, is highly polarized, negatively charged, largely solvent accessible and flexible, all characteristics of IDRs (Fig. 8) [29], [60]. All HEV sequences studied have this IDR, including sequences from avians and rats. As HEV belongs to rubi-like viruses, other members of this family were tested for IDR; only rubivirus and CTV have a region of high polyproline density. Rubivirus possesses two IDR-like domains: one domain associated with its PPR and the other domain located between the macro domain and the endopeptidase (pfam05407) (Fig. 2). It should be noted that the latter domain in rubivirus does not have the high Pro density or positive *dN/dS*. Thus, the PPR is the only region under positive selection in HEV, rubivirus and CTV (Fig. 2).

Positive selection and high homoplastic density in the PPR of HEV genotype 3 and 4 should lead to a highly diverse population of sequences within the PPR. Indeed, the PPR has a high degree of divergence (Fig. 3) and accordingly, has been called the hypervariable region [11]. All these factors taken together with reduction in aa composition complexity should substantially scramble phylogenetic relationships of the PPR from different HEV strains because of the small contribution of many sites to homology in this region. However, an examination of the PPR shows that it has approximately the same degree of phylogenetic signal as seen in most other regions of similar size in ORF1 (Fig. 6). Application of this region to phylogenetic analysis of HEV genotypes and subtypes seems to be as accurate as the use of any other genomic regions [56], [57], [58], [59]. This observation indicates that the observed homoplasy and positive selection are specific to HEV genotypes and subtypes, and occur within the boundaries of the major HEV lineages. Thus, both homoplasy and positive selection most probably reflect recurrent adaptation events experienced by each HEV lineage and are specific to these lineages. These considerations explain the reduced contribution of these factors to the phylogenetic noise at the level of genotypes and subgenotypes.

The PPR does not appear to be required for the replication of rubivirus [18] or HEV [12], [19], [61]. Immediately upstream from the PPR is a region with no known function [10]. Tzeng *et al.* [18] showed that a deletion of this region in rubella virus created a mutant that was unable to replicate. However, Pudupakam *et al.* [12], [19] found that deletions in the PPR did not abolish infectivity of HEV *in vivo* or *in vitro*, although near-complete deletion of the PPR yielded evidence of attenuation. Moreover, Nguyen *et al.* [61] isolated naturally occurring variants isolated in serum and feces from a patient chronically infected with HEV that had deletions in the PPR and macro domain. These observations suggest that the PPR plays a regulatory role in HEV replication, which may be related to proper positioning of structured protein domains [15], [16]. The folding of such hinges is known to regulate the self-assembly of large multiprotein complexes [45] and the PPR may interact with viral and host factors [19].

The recently observed association between deletions in the PPR and variation in levels of viral RNA replication is consistent with the PPR being able to modulate the rate of HEV replication [12]. Ropp *et al.* observed that after extended incubations of 24–36 hours, the HEV ORF1 polyprotein, expressed as a vaccinia recombinant, was cleaved to yield two products *in vivo* [62]. Mutagenesis of Cys in the active site of the putative HEV papain-like protease failed to abolish cleavage, indicating that this protease is not involved in the observed ORF1-protein processing. However, Koonin *et al.* had noted earlier that the papain-like protease motifs found in HEV were atypical [10], which may also explain the result of the mutagenesis experiment. The location of the potential cleavage site [62] appears to be situated near the two putative LM protease cleavage sites identified here (Table 2). This finding may explain, at least in part, the results reported by Pudupakam *et al.* [12], [19].

Sequence analyses of the ORF1 of all HEV genotypes identified the PPR as an IDR (Fig. 4). The identified propensity of the HEV PPR for the disorder-to-order transitions upon interaction with a protein ligand (Fig. 8D) is an important IDR property. The PPR region capable of such transitions is most negatively charged (Fig. 8E) and most prone to protein-ligand binding. As an IDR, the PPR may be involved in regulating viral transcription and translation [45]. IDRs are known to affect protein folding and to bind to large numbers of proteins due to the intrinsically disordered nature of these regions [15], [16]. An examination of LMs found seven putative linear motifs located within the IDR. These include two protease-cleavage sites, three ligand binding sites and two kinase phosphorylation sites (Table 2).

Additionally, the PPR 3D-model was used to predict the occurrence of molecular functional roles using GO annotations. This analysis revealed several sites potentially involved in interactions with many protein ligands (Tables 2 and 4). The motifs include putative peptide cleavage sites, sites modified by enzymes and sites that bind to proteins, nucleotides and metal ions. Such interactions have been shown to contribute to regulation of cellular signal transduction, protein phosphorylation as well as transcription and translation [45]. Thus, these findings suggest that the PPR is involved in protein-protein interactions associated with the regulation of HEV replication. Of further interest are recent reports of isolations of virus/host recombinants found in patients chronically infected with HEV [61], [63]. In both instances a fragment from a human ribosomal protein was inserted in-frame into the PPR, in the first case as a 174-nt (58-aa) insertion from S17 [63] and in the second a 117-nt (39-aa) insertion from S19 [61]. The finding of these insertions provides an additional support to the hypothesis that the PPR has regulatory functions essential for viral adaptation rather than functions critical for viral replication.

In conclusion, the data shown here strongly suggest the role of the PPR in HEV adaptation, including the host-specific adaptation. Being an IDR, the PPR is likely involved in fine tuning of viral replication through protein-protein interactions. Delineation of these interactions will lead to a better understanding of the HEV life cycle and to development of novel anti-viral drugs.

## Supporting Information

Figure S1 **Ramachandran plot.** Backbone dihedral angles ϕ against ψ for the residues in the polyproline peptide structure are plotted on backbone conformational regions of the Ramachandran plot as small black squares, except for Gly, which is shown as black triangles. 98.1% of residues fell into either the most favored regions ([A,B,L] 65.4%, n = 34) or the allowed regions ([a,b,l,p] 32.7%, n = 17). The angles for three residues fell into unfavorable regions (P10, A54 and P67) and are shown as small red squares.
(TIF)

Figure S2 **3D profile quality index.** This figure shows the index for assessment of local environment, packing and hydrophobic energy for the given structure. Values <5 indicate possible local structure or local fold problems. Sequence key: 9 = best, 0 = worst and * indicates a possible problem; PRBLM = problem line markers
(TIF)

Figure S3 **Three-state secondary prediction.** Shown are the probability scores (y-axis), based on PSIPRED, along an 81 aa-long (x-axis) sequence of the HEV genotype 3 PPR (GenBank accession number AB091394) for adopting a helix, strand or coil conformations.
(TIF)

Table S1 **Sequences used in this study.** This table lists the GenBank accession numbers for all sequences examined in this study. A) rubivirus. B) betatetravirus. C) hepevirus. HEV isolated from Chinese rabbits is tentatively classified as genotype 3, and denoted as 3* in the table. HEV isolated from avians, Japanese wild boars and rats is unclassified, and denoted with a, w or r, respectively, in the type column. HQ731075 is the cutthroat trout virus.
(DOC)
