# Supplementary figures and images for: The Hepatitis E Virus Polyproline Region Is Involved in Viral Adaptation
Source: PLoS One. 2012 Apr 24;7(4):e35974. doi: 10.1371/journal.pone.0035974 (PMC3335810; doi:10.1371/journal.pone.0035974)

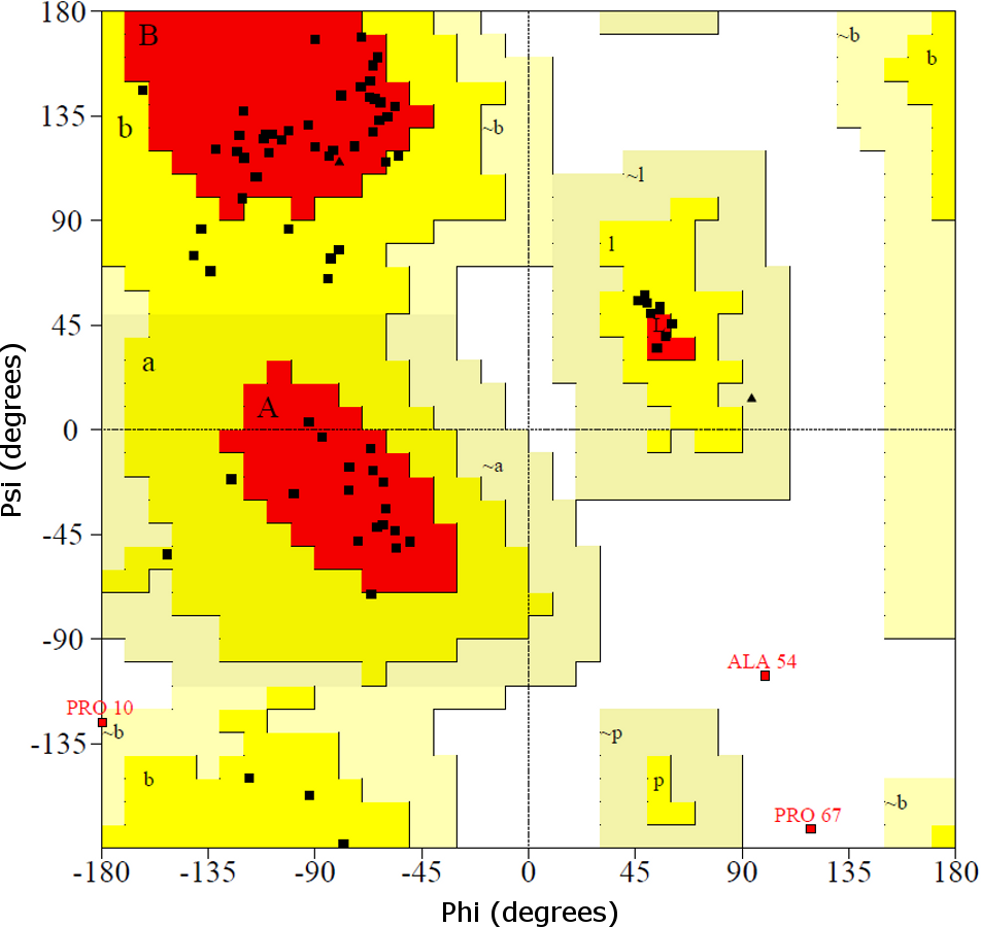

Supplement: Figure S1 — Ramachandran plot. Backbone dihedral angles ϕ against ψ for the residues in the polyproline peptide structure are plotted on backbone conformational regions of the Ramachandran plot as small black squares, except for Gly, which is shown as black triangles. 98.1% of residues fell into either the most favored regions ([A,B,L] 65.4%, n = 34) or the allowed regions ([a,b,l,p] 32.7%, n = 17). The angles for three residues fell into unfavorable regions (P10, A54 and P67) and are shown as small red squares. (TIF) [file pone.0035974.s001.tif]

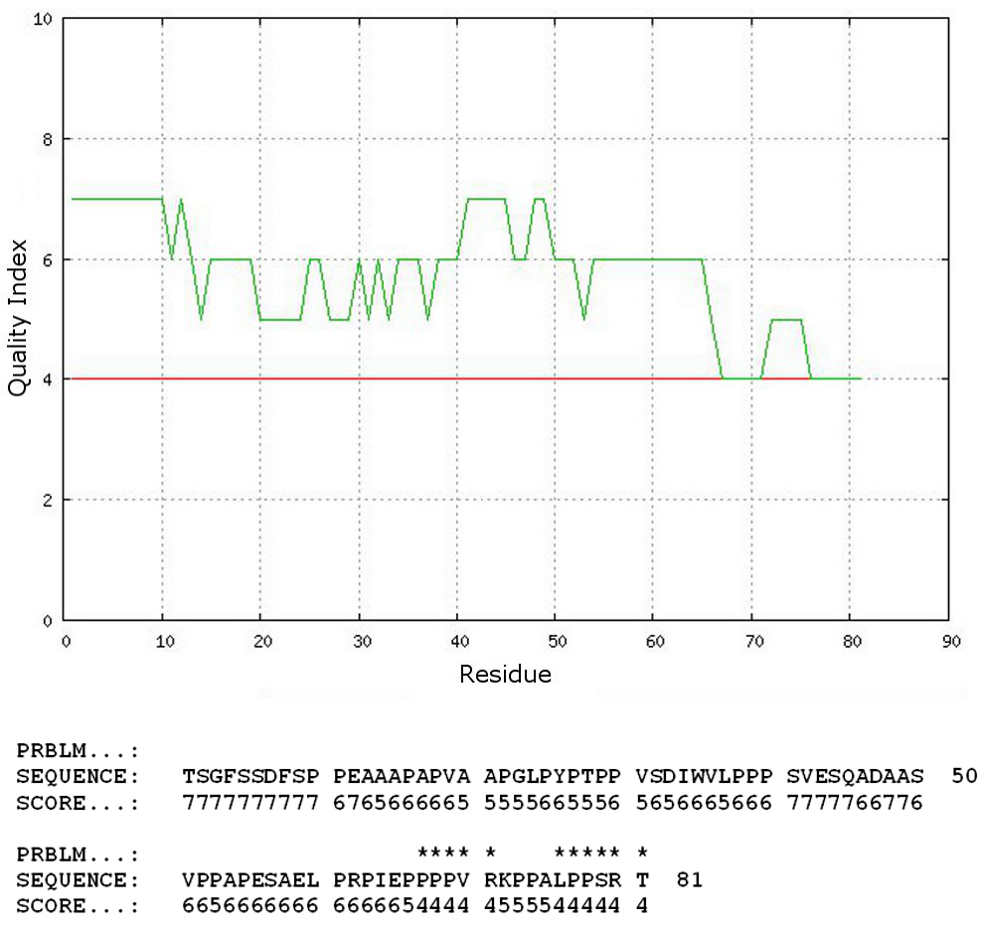

Supplement: Figure S2 — 3D profile quality index. This figure shows the index for assessment of local environment, packing and hydrophobic energy for the given structure. Values <5 indicate possible local structure or local fold problems. Sequence key: 9 = best, 0 = worst and * indicates a possible problem; PRBLM = problem line markers (TIF) [file pone.0035974.s002.tif]

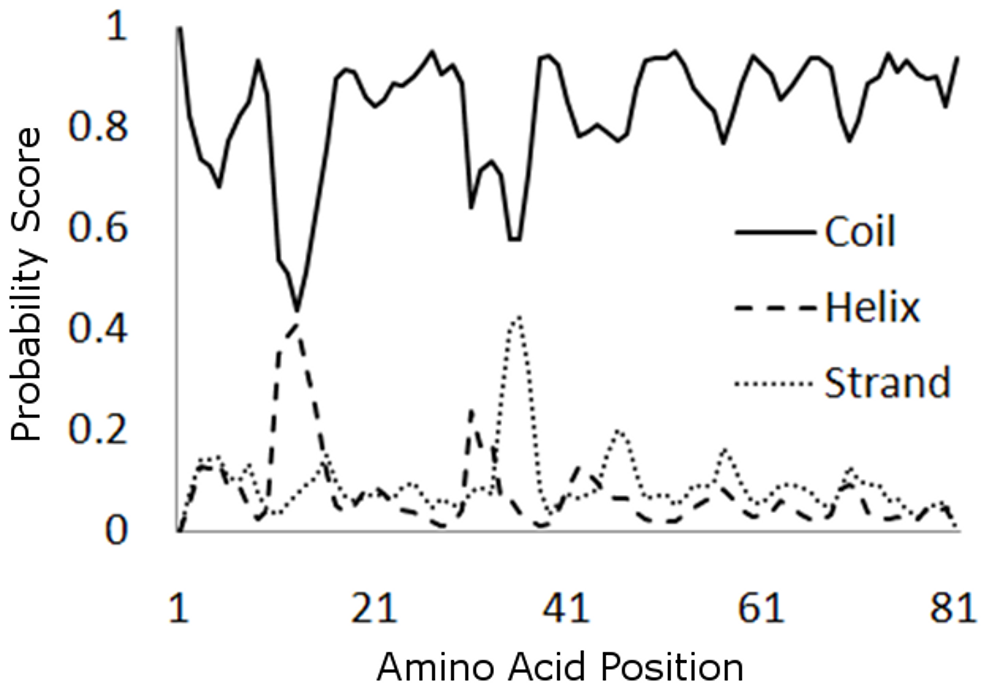

Supplement: Figure S3 — Three-state secondary prediction. Shown are the probability scores (y-axis), based on PSIPRED, along an 81 aa-long (x-axis) sequence of the HEV genotype 3 PPR (GenBank accession number AB091394) for adopting a helix, strand or coil conformations. (TIF) [file pone.0035974.s003.tif]
