# Supplementary material for: The Hepatitis E Virus Polyproline Region Is Involved in Viral Adaptation
Source: PLoS One. 2012 Apr 24;7(4):e35974. doi: 10.1371/journal.pone.0035974 (PMC3335810; doi:10.1371/journal.pone.0035974)
Supplement: Table S1 — Sequences used in this study. This table lists the GenBank accession numbers for all sequences examined in this study. A) rubivirus. B) betatetravirus. C) hepevirus. HEV isolated from Chinese rabbits is tentatively classified as genotype 3, and denoted as 3* in the table. HEV isolated from avians, Japanese wild boars and rats is unclassified, and denoted with a, w or r, respectively, in the type column. HQ731075 is the cutthroat trout virus. (DOC) [file pone.0035974.s004.doc]

A.

| AB047329 | AF188704 | AY258323 | DQ085341 | DQ388280 | L78917 |
| --- | --- | --- | --- | --- | --- |
| AB047330 | AF435865 | DQ085338 | DQ085342 | DQ388281 | M15240 |
| AB222608 | AF435866 | DQ085339 | DQ085343 | FJ211587 | NC_001545 |
| AB222609 | AY258322 | DQ085340 | DQ388279 | FJ211588 |  |

B.

| AF102884 | AF461742 | GU991616 | NC_001990 | NC_003412 | NC_014126 |
| --- | --- | --- | --- | --- | --- |

C.

| ID | type |  | ID | type |  | ID | type |  | ID | type |
| --- | --- | --- | --- | --- | --- | --- | --- | --- | --- | --- |
| AB073912 | 3 |  | AB236320 | 3 |  | AB443624 | 3 |  | EU360977 | 3 |
| AB074915 | 4 |  | AB246676 | 3 |  | AB443625 | 3 |  | EU366959 | 4 |
| AB074917 | 4 |  | AB248520 | 3 |  | AB443626 | 3 |  | EU375463 | 3 |
| AB074918 | 3 |  | AB248521 | 3 |  | AB443627 | 3 |  | EU495148 | 3 |
| AB074920 | 3 |  | AB248522 | 3 |  | AB480825 | 4 |  | EU676172 | 4 |
| AB080575 | 4 |  | AB253420 | 4 |  | AB481226 | 3 |  | EU723512 | 3 |
| AB089824 | 3 |  | AB290312 | 3 |  | AB481227 | 4 |  | EU723513 | 3 |
| AB091394 | 3 |  | AB291951 | 3 |  | AB481228 | 3 |  | EU723514 | 3 |
| AB091395 | 4 |  | AB291952 | 3 |  | AB481229 | 3 |  | EU723515 | 3 |
| AB097811 | 4 |  | AB291953 | 3 |  | AB521805 | 4 |  | EU723516 | 3 |
| AB097812 | 4 |  | AB291954 | 3 |  | AB521806 | 4 |  | FJ426403 | 3 |
| AB099347 | 4 |  | AB291955 | 3 |  | AB573435 | w |  | FJ426404 | 3 |
| AB108537 | 4 |  | AB291956 | 3 |  | AB602441 | w |  | FJ457024 | 1 |
| AB161717 | 4 |  | AB291957 | 3 |  | AF028091 | 1 |  | FJ527832 | 3 |
| AB161718 | 4 |  | AB291958 | 3 |  | AF051830 | 1 |  | FJ610232 | 4 |
| AB161719 | 4 |  | AB291959 | 4 |  | AF060668 | 3 |  | FJ653660 | 3 |
| AB189070 | 3 |  | AB291960 | 3 |  | AF060669 | 3 |  | FJ705359 | 3 |
| AB189071 | 3 |  | AB291961 | 3 |  | AF076239 | 1 |  | FJ763142 | 4 |
| AB189072 | 3 |  | AB291962 | 3 |  | AF082843 | 3 |  | FJ906895 | 3* |
| AB189073 | 3 |  | AB291963 | 3 |  | AF185822 | 1 |  | FJ906896 | 3* |
| AB189074 | 3 |  | AB291964 | 4 |  | AF455784 | 3 |  | FJ956757 | 3 |
| AB189075 | 3 |  | AB291965 | 4 |  | AF459438 | 1 |  | FJ998008 | 3 |
| AB193176 | 4 |  | AB291966 | 4 |  | AJ272108 | 4 |  | GU119960 | 4 |
| AB193177 | 4 |  | AB291967 | 4 |  | AM943646 | a |  | GU119961 | 4 |
| AB193178 | 4 |  | AB291968 | 4 |  | AM943647 | a |  | GU188851 | 4 |
| AB197673 | 4 |  | AB301710 | 3 |  | AP003430 | 3 |  | GU206559 | 4 |
| AB197674 | 4 |  | AB362839 | 3 |  | AY115488 | 3 |  | GU345042 | r |
| AB200239 | 4 |  | AB362840 | 3 |  | AY204877 | 1 |  | GU345043 | r |
| AB220971 | 4 |  | AB362841 | 3 |  | AY230202 | 1 |  | GU361892 | 4 |
| AB220972 | 4 |  | AB362842 | 3 |  | AY535004 | a |  | GU937805 | 3* |
| AB220973 | 4 |  | AB362843 | 3 |  | AY594199 | 4 |  | GU954430 | a |
| AB220974 | 4 |  | AB369687 | 3 |  | AY723745 | 4 |  | L25595 | 1 |
| AB220975 | 4 |  | AB369688 | 4 |  | D10330 | 1 |  | M73218 | 1 |
| AB220976 | 4 |  | AB369689 | 3 |  | D11092 | 1 |  | M74506 | 2 |
| AB220977 | 4 |  | AB369690 | 4 |  | D11093 | 1 |  | M80581 | 1 |
| AB220978 | 4 |  | AB369691 | 3 |  | DQ279091 | 4 |  | M94177 | 1 |
| AB220979 | 4 |  | AB425830 | 3 |  | DQ450072 | 4 |  | X98292 | 1 |
| AB222182 | 3 |  | AB425831 | 3 |  | DQ459342 | 1 |  | X99441 | 1 |
| AB222183 | 3 |  | AB437317 | 3 |  | EF077630 | 4 |  |  |  |
| AB222184 | 3 |  | AB443623 | 3 |  | EF570133 | 4 |  |  |  |
